# Ninjinyoeito Improves Behavioral Abnormalities and Hippocampal Neurogenesis in the Corticosterone Model of Depression

**DOI:** 10.3389/fphar.2018.01216

**Published:** 2018-10-26

**Authors:** Kenta Murata, Nina Fujita, Ryuji Takahashi, Akio Inui

**Affiliations:** ^1^Kampo Research Laboratories, Kracie Pharma, Ltd., Tokyo, Japan; ^2^Pharmacological Department of Herbal Medicine, Kagoshima University Graduate School of Medical and Dental Sciences, Kagoshima, Japan

**Keywords:** ninjinyoeito, depression, neurogenesis, corticosterone, neuronal progenitor cell

## Abstract

Ninjinyoeito (NYT), a traditional Chinese medicine consisting of 12 herbs, is designed to improve fatigue, cold limbs, anorexia, night sweats, and anemia. Recently, NYT was reported to improve cognitive outcome and depression in patients with Alzheimer’s disease. However, little is known about how NYT alleviates depression and cognitive dysfunction. In this study, we investigated the effects and mechanisms of NYT in a corticosterone (CORT)-induced model of depression. Chronic NYT treatment ameliorated the depressive-like behaviors induced by CORT treatment in three types of behavioral tests. In addition, chronic NYT treatment also improved memory disruptions induced by CORT in both the Y-maze and novel object recognition tests, without affecting locomotor activity. Furthermore, we also showed that NYT treatment attenuated the CORT-induced reduction in cell proliferation and immature neuronal cell numbers in mouse hippocampal dentate gyrus. These results suggest that NYT has therapeutic effects on CORT-induced behavioral abnormalities and inhibition of hippocampal neurogenesis.

## Introduction

Depression is one of the most common mood disorders in modern society. It is estimated that about 1% of the population will be affected by major depression at least once during their lifetime (Rao et al., 2004). Although extensive studies have led to various hypotheses regarding the molecular mechanisms underlying depression, the pathogenesis of depression remains to be fully clarified. One hypothesis concerns the hypothalamic-pituitary-adrenal (HPA)-axis, which is well known to play a critical role in the pathogenesis of mood disorders (de Kloet et al., 2005, 2006). Under normal conditions, glucocorticoid levels in blood are sensitively regulated by the HPA-axis via negative feedback. However, in stressful situations, persistently high concentrations of glucocorticoids in the blood causes dysregulation of the HPA-axis, induces atrophy in the central nervous system, and even aggravates depression (Murphy, 1997; Sapolsky, 2000). Based on these findings, the chronic corticosterone (CORT) exposure model is widely used to induce depressive-like behavioral and neurochemical changes in rodents (Gourley et al., 2008). Recent work suggests that elevated glucocorticoid levels induces behavioral disorder and decreases cell proliferation in the hippocampal dentate gyrus, and that these changes are reversed by chronic antidepressant treatment. In addition, the efficacy of some antidepressants, such as fluoxetine, is abrogated by X-irradiation, which inhibits hippocampal neurogenesis (David et al., 2009). Thus, improving hippocampal neurogenesis is considered to be one of the most important strategies for the development of antidepressant medicines.

Ninjinyoeito (NYT), a traditional Chinese medicine described in Ho-chi-chü-fang, is designed to improve fatigue, cold limbs, anorexia, night sweats, and anemia. For that reason, NYT has been administered to elderly people in Traditional Oriental medicine. NYT is composed of 12 herbs: ginseng, astragalus root, angelica root, rehmannia root, atractylodes root, poria sclerotium, peony root, cinnamon bark, citrus unshiu peel, polygala root, schisandra fruit, and glycyrrhiza. In basic research, NYT was reported to improve memory impairments and the reduction of serotonin and 5-hydroxyindole-3-acetic in the olfactory bulb lesion mouse model (Song et al., 2001). NYT was also reported to improve demyelination and increase the number of oligodendrocytes in aged or cuprizone-treated rodents (Kobayashi et al., 2003; Seiwa et al., 2007). Another report suggested that NYT increased nerve growth factor in astrocytes (Yabe et al., 2003). In addition to basic research, a recent clinical trial revealed that NYT treatment improved cognitive outcome and Alzheimer’s disease-related depression in patients with Alzheimer’s disease (Kudoh et al., 2016). Although the action of NYT in the central nervous system is being clarified by some animal model studies, little is known about how NYT alleviates depression and cognitive disorder.

Here, we investigated how NYT may improve depression and cognitive disorder using the chronic CORT-induced model of depression.

## Materials and Methods

### Animals

Five-week-old male C57BL/6 mice (SLC, Shizuoka, Japan) were used in these experiments. Animals were housed at 24 ± 2°C under a 12-h light–dark cycle (lights on from 8:00 to 20:00) with *ad libitum* access to food and water. Behavioral experiments were performed between 9:00 and 18:00. All efforts were made to minimize both the suffering of and the number of animals used. The experimental protocol was reviewed and approved by the Experimental Animal Care Committee of Kracie Pharma, Ltd. (Toyama, Japan).

### Plant Materials and Preparation of the Extract

Ninjinyoeito is composed of twelve dried medical herbs, including rehmannia root, Japanese angelica root, atractylodes rhizome, poria sclerotium, ginseng, cinnamon bark, polygala root, peony root, citrus unshiu peel, astragalus root, glycyrrhiza, and schisandra fruit (Table 1), and is supplied by Kracie Pharma, Ltd. as a dried extract powder. Each plant material was identified by external morphology and authenticated by marker compounds of plant specimens according to the method of Japanese Pharmacopeia and our company’s standard. The extract powder (lot no. 15112017) was suspended in distilled water immediately before use and was administered orally at a dose of 500 or 1000 mg/kg body-weight/day.

**Table 1:** Medical herb composition of NYT.

Common name	Botanical name	weight (g)
Rehmannia Root	Rehmannia glutinosa (Gaertn.) Libosch. ex Fisch. & C.A. Mey.	4
Japanese Angelica root	Angelica acutiloba (Siebold & Zucc.) Kitag.	4
Atractylodes Rhizome	Atractylodes japonica Koidz. ex Kitam.	4
Poria Sclerotium	Wolfiporia cocos Ryvarden et Gilbertson	4
Ginseng	Panax ginseng C.A.Mey.	3
Cinnamon Bark	Cinnamomum cassia (L.) J.Presl	2.5
Polygala Root	Polygala tenuifolia Willd.	2
Peony Root	Paeonia lactiflora Pall.	2
Citrus Unshiu Peel	Citrus unshiu Markowicz	2
Astragalus Root	Astragalus membranaceus (Fisch.) Bunge	1.5
Glycyrrhiza	Glycyrrhiza uralensis Fisch.	1
Schisandra Fruit	Schisandra chinensis (Turcz.) Baill.	1

### High-Performance Liquid Chromatography Analysis of NYT

Ninjinyoeito extract was mixed and shaken with 50% MeOH and the supernatant was subjected to high-performance liquid chromatography (HPLC) analysis. The three-dimensional HPLC profile of NYT was obtained using a Shimazu LC-30AD liquid chromatography equipped with an SPD-M30A detector with scanning for a range of 230–400 nm and a reversed-phase column (Shim-pack XR-ODSIII, 2.0 mm i.d. × 50 mm, 1.6 mm, Column temperature: 40°). The column was equipped with solvent A (0.1% formic acid in acetonitrile) and solvent B (0.1% formic solution), and the ratio of solvent A was increased by 5% over 16 min, 70% over 1 min, and 5% over 1 min, with a flow rate at 0.5 mL/min.

### Drug Treatment

Five-week-old male C57BL6 mice were used for chronic oral CORT exposure. Mice were divided into 5 groups: control group (*n* = 10), CORT-treated group (*n* = 10), CORT + NYT (500 or 1000 mg/kg)-treated group (*n* = 10), CORT + imipramine-treated group (*n* = 10). Mice were administered CORT (100 μg/mL; Sigma-Aldrich, St. Louis, MO, United States) in place of drinking water for 14 days. Animal were weaned with 50 μg/mL CORT for 3 days and then with 25 μg/mL CORT for 3 days to allow for gradual recovery of endogenous corticosterone secretion. NYT (500 or 1000 mg/kg/day) was orally administered once daily from day 21 to day 49. As a positive control, imipramine (10 mg/kg/day, intraperitoneally (i.p.); Wako Pure Chemical, Osaka, Japan) was administered once daily. Subsequent behavioral tests were performed on days 50–64 and brain samples were collected on day 65. On the days behavioral tests were performed, the drugs were administered 30 min before the tests. A 5-bromo-2-deoxyuridine (BrdU) solution (50 mg/kg/day, i.p.; Sigma-Aldrich) was administered from day 15 to day 19.

### Open Field Test

Each mouse was placed in the periphery of the open field apparatus (width 30 cm × length 30 cm × height 30 cm). The total distance traveled in the arena and the time spent in the center zone (width 15 cm × length 15 cm) was recorded for 10 min using a video tracking system, ANY-maze (Muromachi Kikai Co., Ltd., Japan).

### Tail Suspension Test

We performed the tail suspension test as described in a previous report (Can et al., 2012). Briefly, the tails of mice were suspended with a piece of adhesive tape 50 cm above the floor with climbstoppers (clear plastic cylinder, 3 cm length, 1 cm outside diameter, 0.5 cm inside diameter), and animal behavior was recorded for 6 min. As a test parameter, the latency to immobility and the total immobility time in the last 4 min were measured manually in a blinded manner. Small movements that were confined to the front legs, but without the involvement of the hind legs, were counted as immobility. Additionally, oscillations and pendulum-like swings that were due to the momentum gained during the earlier mobility bouts were also counted as immobility. The latency to immobility was determined as the time required for the mouse to first cease all movement for > 5 s.

### Forced Swim Test

Mice were placed in a glass cylinder (height, 30 cm; diameter, 15 cm) filled with water (23 ± 2°C) to a 15-cm depth for 6 min. Mice were judged to be immobile when they floated passively in the water, making only small movements to maintain their body balance or to keep their heads above the water. As a test parameter, the latency to immobility and the total immobility and mobility time during the last 4 min were measured manually in a blinded manner. The latency to immobility was determined as the time required for the mouse to first cease all movement for > 2 s.

### Sucrose Preference Test

Animals were habituated to drinking water from two bottles for 2 days. Mice were deprived of water for 14 h before the test, and the test was carried out on the following morning at 10:00. In the sucrose preference test, two pre-weighed bottles [one containing tap water and the other containing a 1% (w/v) sucrose solution] were presented to each animal for 4 h. The position of the water and sucrose bottles (left or right) was switched every 2 h. The bottles were weighed again, and the weight difference represented the animal’s intake from each bottle. The sum of the volume of water plus sucrose intake was defined as the total volume intake, and sucrose preference was expressed as the percentage of sucrose intake relative to the total intake.

### Y-Maze Test

The Y-maze apparatus has three arms at 120° angles (width 8 cm × length 30 cm × height 15 cm) extending from a central space (8 × 8 cm). Each mouse was placed in one arm and allowed to explore freely for 8 min to assess the rate of spontaneous alternation, defined as consecutive entries into three different arms without repetition. The spontaneous alternation percentage was calculated by the equation [successive entries/(total arm entries -1) × 100].

### Novel Object Recognition Test

Each mouse was placed in the open-field apparatus after being habituated to the apparatus (without objects) for 15 min prior to the training session. At the end of each trial, the mouse was removed from the arena, and the arena was cleaned with 70% ethanol solution and dried with paper towels. Object recognition was scored by the amount of time spent with each object (defined as time spent with the nose directed to the object and/or with forelimbs touching the object). In the training session (T1: 10 min), two similar objects (left and right: cubes) were placed in a symmetrical position 5 cm away from the wall. In the retention session (T2: 10 min), two dissimilar objects were presented [one a cube (familiar), as before, and the other a new object, a cylinder (novel)]. The time spent exploring each object was recorded during T1 and T2. All mice were tested with a 2-h interval between T1 and T2.

### Cell Culture

Adult mouse hippocampal progenitor/stem cells (NPCs) were isolated from the dentate gyrus of 5-week-old C57BL/6 mice as previously described (Babu et al., 2011). Briefly, mice were euthanized with isoflurane and decapitated with surgical scissors. The area of the dentate gyrus was isolated under the microscope, and the tissue was incubated with an enzyme mixture (2.5 U/mL papain, 1 U/mL dispase, 250 U/mL DNase) for 20 min at 37°C and triturated to obtain a single-cell suspension. NPCs were isolated from the cell mixture by using 22% vol/vol Percoll solution (GE Healthcare Japan, Tokyo, Japan) and centrifugation. Isolated NPCs were re-suspended in Neurobasal A (Thermo Fisher Scientific, Waltham, MA, United States) supplemented with 2% B27 (Sigma-Aldrich), 2 mM glutamine, 20 ng/ml epidermal growth factor (EGF), and 20 ng/mL fibroblast growth factor (FGF)-2 (Miltenyi Biotec, Bergisch Gladbach, Germany). Cells were dispersed and passaged weekly and cells passaged 2–4 times were used for experiments.

### Cell Proliferation Assay

Isolated NPCs were seeded at 1.5 × 10^3^ cells/well in 96-well plates coated with poly-D-lysine (PDL)/laminin and then incubated for 24 h. NPCs were cultured for 72 h in the presence of 20 μM CORT and/or NYT in proliferation solution. The synthetic nucleotide BrdU (10 μM; Sigma-Aldrich) was added to the medium 4 h before treatment cessation. NPCs were fixed with 4% paraformaldehyde for 30 min at room temperature.

### Immunohistochemistry

Mice were anesthetized with isoflurane and perfused with saline until the outflow was clear, then immediately perfused with 4% paraformaldehyde for 10 min. Brains were removed and post-fixed in the same fixative for 24 h at 4°C. Paraffin-embedded tissue (thickness, 10 μm) was used for immunohistochemical identification of Ki67-, BrdU-, doublecortin (DCX)-, and glial fibrillary acidic protein (GFAP)-positive cells. Formalin-fixed, paraffin-embedded tissue sections were deparaffinized and hydrated. For BrdU immunostaining, the sections were incubated with 2N hydrochloric acid (HCl) for 30 min. Endogenous peroxidase was inhibited by incubation with freshly prepared 3% hydrogen peroxide (H_2_O_2_) with methanol. The sections were treated with citrate buffer at 121°C for 10 min. Non-specific staining was blocked with 5% goat serum for 60 min at room temperature. The sections were then incubated with rabbit polyclonal anti-DCX antibody (1:400; Abcam, Cambridge, MA, United States), rabbit polyclonal anti-GFAP antibody (1:200; Cell Signaling Technology, Danvers, MA, United States), rabbit polyclonal anti-Ki67 antibody (1:400; Abcam), or mouse monoclonal anti-BrdU (1:400, Cell Signaling Technology) at 4°C. Staining was developed with diaminobenzidine (Sigma-Aldrich) substrate and the cell density was calculated by dividing the number of cells counted by the volume of the counted area and averaged per animal. For the *in vitro* study, NPCs incorporating BrdU were incubated with 2 N HCl for 30 min at 37°C. NPCs were washed in phosphate buffered saline (PBS) and blocked with 10% normal goat serum for 60 min at room temperature. Subsequently, the NPCs were incubated overnight at 4°C with mouse monoclonal anti-BrdU antibody (1:1000; Cell Signaling Technology, Danvers, MA, United States). After being washed in PBS, the cells were reacted with Alexa Fluor 594 goat anti-mouse IgG (1:1000; Invitrogen, Carlsbad, CA, United States) for 1 h at room temperature. Nuclei were stained with Hoechst 33342 (1:2000; Invitrogen) for 20 min in the dark. The proliferation rate was calculated by dividing the number of BrdU-positive cells by the total cell number.

### Statistical Analysis

**FIGURE 1:**
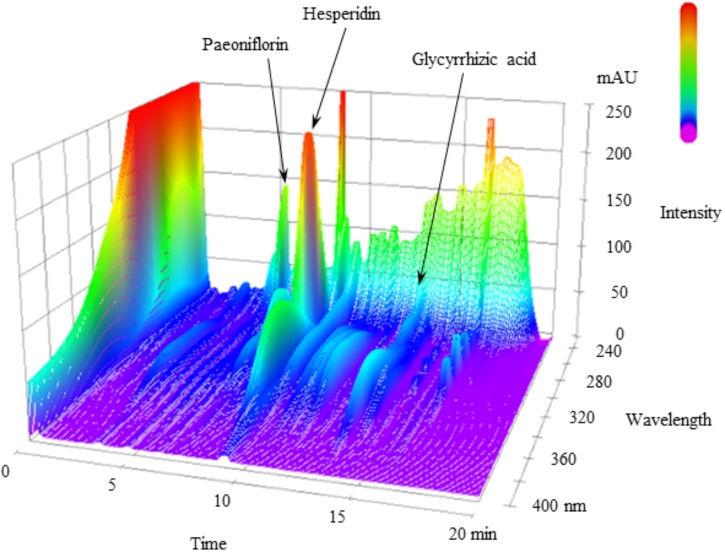
3D-HPLC profile of NYT. Each chemical marker (paeoniflorin, hesperidin, and glycyrrhizic acid) in the HPLC profile was identified by comparison with retention times and UV spectra (230–400 nm) of their reference standards.

**FIGURE 2:**
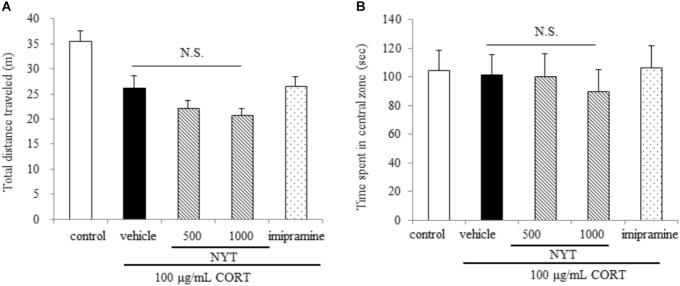
Effect of NYT on locomotor activity and anxiety-like behavior in CORT-treated mice. Effect of repeated treatment with NYT on the total distance traveled **(A)** and the time spent in the center zone **(B)** in the open field test. Data are expressed as mean ± SEM (*n* = 9–10).

**FIGURE 3:**
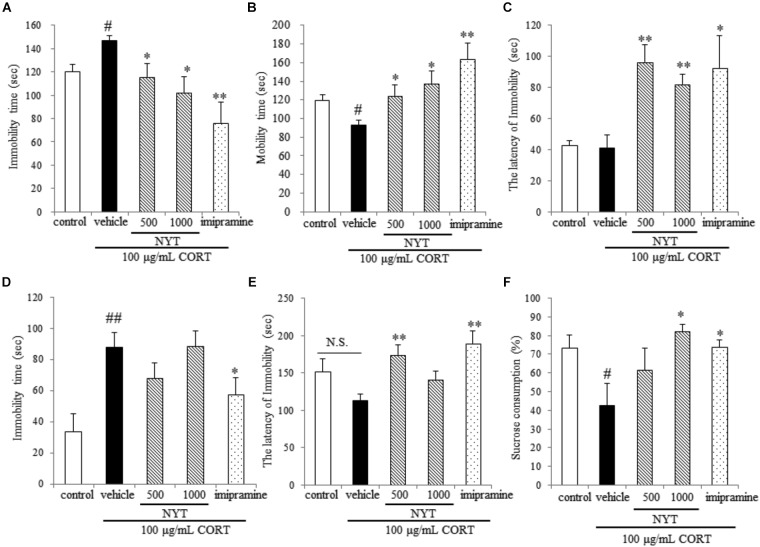
Effect of NYT on depressive-like behaviors in CORT-treated mice. Effect of repeated treatment with NYT on the immobility time **(A)**, mobility time **(B)**, and latency to immobility **(C)** in the tail suspension test. Effect of repeated treatment with NYT on the duration **(D)** and the latency to immobility **(E)** in the forced swim test. **(F)** Effect of repeated treatment with NYT on the sucrose consumption rate in the sucrose preference test. Data are expressed as the mean ± SEM (*n* = 9–10). ^#^*p* < 0.05, ^##^*p* < 0.01 vs. the control group; ^∗^*p* < 0.05, ^∗∗^*p* < 0.01 vs. the vehicle-treated group, Student’s *t*-test.

**FIGURE 4:**
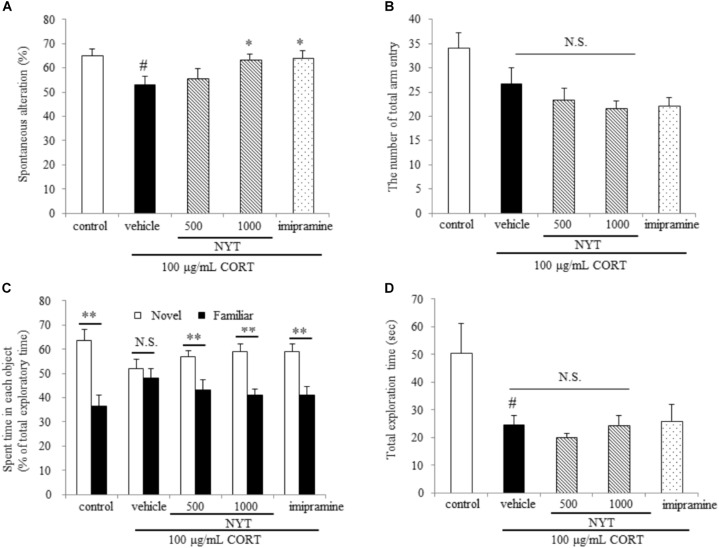
Effect of NYT on memory impairment in CORT-treated mice. Effect of repeated treatment with NYT on spontaneous alternations **(A)** and the number of total arm entries **(B)** in the Y-maze test. #*p* < 0.05 vs. the control group; ^∗^*p* < 0.05 vs. the vehicle-treated group, Student’s *t*-test. Effect of repeated treatment with NYT on the time spent with each object in the novel recognition test and total exploration time **(C)** is the time spent with each object and **(D)** is the total exploration time in novel object recognition test. Data are expressed as the mean ± SEM (*n* = 9–10). ^∗∗^*p* < 0.01 vs. the time spent with a familiar object, Student’s *t*-test.

**FIGURE 5:**
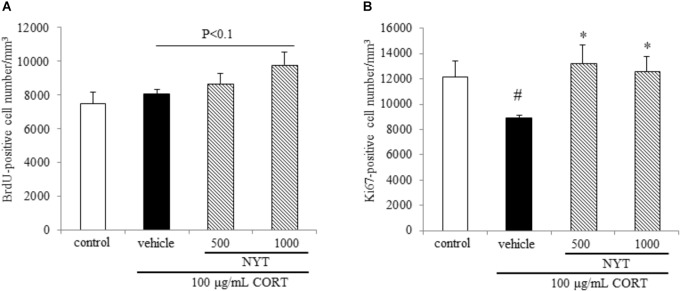
Effect of NYT on cell survival and proliferation in the mouse hippocampus. Effect of repeated treatment with NYT on cell survival and cell proliferation in the dentate gyrus of the mouse hippocampus. Quantitative analyses of the density of BrdU-positive cells **(A)** and Ki67-positive cells **(B)** in the hippocampus. Data are expressed as the mean ± SEM (*n* = 5). ^#^*p* < 0.05 vs. the control group; ^∗^*p* < 0.05 vs. the vehicle-treated group, Student’s *t*-test.

**FIGURE 6:**
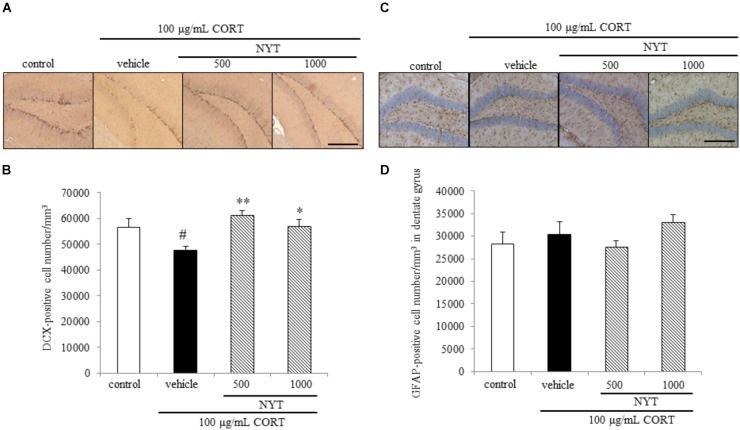
Effect of NYT on the number of immature neurons and astrocytes in the mouse hippocampus. Effect of repeated treatment with NYT on the density of DCX- and GFAP-positive cells (cells/mm^3^) in the dentate gyrus of the mouse hippocampus. **(A)** Representative image of DCX staining in the hippocampus. Scale bar = 200 μm. **(B)** Quantitative analyses of the total number of DCX-positive cells in the hippocampus. **(C)** Representative image of GFAP staining in the hippocampus. Scale bar = 200 μm. **(D)** Quantitative analyses of the total number of GFAP-positive cells in the hippocampus. Data are expressed as the mean ± SEM (*n* = 3–4). ^#^*p* < 0.05 vs. the control group; ^∗^*p* < 0.05, ^∗∗^*p* < 0.01 vs. the vehicle-treated group, Student’s *t*-test.

All data are expressed as mean ± standard error of the mean (SEM). Statistical comparisons were performed using a one-way analysis of variance (ANOVA) followed by a Student’s *t-*test or Dunnett’s test. The Student’s *t*-test was used to analyze the differences between the two groups in Figures 1–5. Dunnett’s *post hoc* test was used for the results shown in Figure 6. Differences with *p* < 0.05 were considered statistically significant.

## Results

### HPLC Analysis of NYT

Figure 1 shows a 3D-HPLC profile of NYT along with a chemical analysis. Chemical makers, such as paeoniflorin, hesperidin, and glycyrrhizic acid, were used for quality control.

### Effect of Treatment With NYT on CORT-Induced Behavioral Abnormalities

To investigate the effect and mechanism of action of NYT on depressive-like behaviors, we used the chronic CORT-induced model of depression, which is widely used for depression research. To induce the depressive-like symptoms, C57BL/6 mice were treated with CORT in their drinking water for 3 weeks. After CORT treatment finished, mice were treated with NYT or imipramine for 4 weeks, and we examined whether NYT improved CORT-induced behavioral abnormalities by performing behavioral tests.

We first evaluated the effect of a 4-week treatment with NYT on spontaneous locomotor activity and anxiety-related behavior in mice. For this purpose, we performed the open field test for 10 min. In the open field test, chronic exogenous CORT treatment tended to decrease the total distance traveled, but this effect was not significant. Long-term CORT treatment had no effect on the time spent in the center zone. In addition, NYT treatment had no effect on either the total distance traveled or the time spent in the center zone compared with the vehicle-treated group. Treatment with imipramine also did not have any effect on either the total distance traveled or the time spent in the center zone (Figures 2A,B).

We next evaluated the effect of a 4-week treatment with NYT on CORT-induced depressive-like behavior. For this purpose, we used three behavioral tests: the tail suspension test, the forced swim test, and the sucrose preference test. In the tail suspension test, chronic exogenous CORT treatment significantly increased the immobility time compared with the control group; this increase continued for 4 weeks after completion of the CORT treatment. In addition, a 4-week treatment with NYT and imipramine significantly decreased immobility time compared with the vehicle-treated group (Figure 3A). The latency to immobility was also increased in the NYT and imipramine-treated groups, but there was no difference between the control and CORT-treated groups (Figure 3C). In the forced swim test, chronic exogenous CORT treatment significantly increased immobility time compared with the control group; this increase continued for 4 weeks after completion of the CORT treatment. However, chronic exogenous CORT treatment had no effect on the latency to immobility compared with the control group. NYT (500 mg/kg) increased the latency to immobility, but NYT did not improve total immobility time (Figures 3D,E). In the sucrose preference test, chronic exogenous CORT treatment significantly decreased the sucrose consumption rate compared with the control group. In addition, treatment with NYT and imipramine significantly improved the sucrose consumption rate (Figure 3F).

We further evaluated the effect of NYT on memory disruption in the CORT-induced depression model. For this purpose, we used two behavioral tests: the Y-maze test and the novel object recognition test. In the Y-maze test, chronic exogenous CORT treatment significantly decreased spontaneous alternations, but the number of total arm entries was unchanged compared with the control group. On the other hand, a 4-week treatment with NYT significantly improved spontaneous alternations compared with the vehicle-treated group (Figures 4A,B). In the novel object recognition test, mice treated with CORT could not distinguish between familiar and novel objects but the control and NYT-treated groups spent more time with the novel object. The total exploration time was significantly decreased by CORT treatment, but NYT treatment did not improve this when compared with the vehicle-treated group (Figures 4C,D).

These results indicate that NYT improves the depressive-like behaviors and memory disruption induced by CORT treatment without affecting locomotor activity.

### Effect of NYT on CORT-Induced Inhibition of Hippocampal Neurogenesis in the CORT-Induced Depression Model

To investigate the mechanisms underlying the effects of NYT in this depression model, we focused on adult hippocampal neurogenesis, which contributes to the action of antidepressants. To evaluate the effect of NYT on hippocampal neurogenesis, we measured the BrdU-, Ki67-, DCX-, and GFAP-positive cell numbers in the mouse hippocampal dentate gyrus 4 weeks after NYT treatment started. To evaluate the effect on cell survival, we first measured the BrdU-positive cell number in the dentate gyrus. The mice were treated with 50 mg/kg BrdU for 5 days before NYT treatment started. Chronic CORT treatment did not change the number of BrdU-positive cells in the dentate gyrus compared with the control group. In addition, 1000 mg/kg NYT treatment tended to increase the number of BrdU-positive cells in the dentate gyrus compared with the vehicle-treated group, but this increase was not statistically significant (Figure 5A). This result indicates that NYT treatment did not affect the cell survival rate in the mouse hippocampal dentate gyrus.

To evaluate the effect on cell proliferation, we next measured the number of endogenous mitotic marker Ki67-positive cells in the dentate gyrus. As a result, chronic CORT treatment continued to decrease the number of Ki67-positive cells for 6 weeks after completion of the CORT treatment. The decrease in the Ki67-positive cell number was improved by 4 weeks of NYT treatment (Figure 5B). This result indicates that NYT treatment improved cell proliferation in the mouse hippocampus.

We also measured DCX- and GFAP-positive cell numbers in the dentate gyrus. DCX is broadly expressed in neuroblasts and immature neurons in neurogenic regions of the adult brain (Gleeson et al., 1999; Brown et al., 2003). GFAP is an intermediate filament expressed in astrocytes. GFAP is also expressed in the neural stem cells with radial glia-like morphology in the subgranular zone of dentate gyrus, so we counted the GFAP positive cell number in dentate gyrus except subgranular zone. In agreement with other reports (Hill et al., 2015), chronic CORT treatment reduced the number of DCX-positive cells in the dentate gyrus of the adult mouse hippocampus. The decrease in the DCX-positive cell number was improved by 4 weeks of NYT treatment (Figures 6A,B). On the other hand, chronic CORT and NYT treatment did not change the number of GFAP-positive cells in the dentate gyrus (Figures 6C,D). These results suggest that NYT treatment attenuated the CORT-induced inhibition of hippocampal neurogenesis.

### Effect of NYT on CORT-Induced Inhibition of NPC Proliferation as Assessed Using an *in vitro* Assay

**FIGURE 7:**
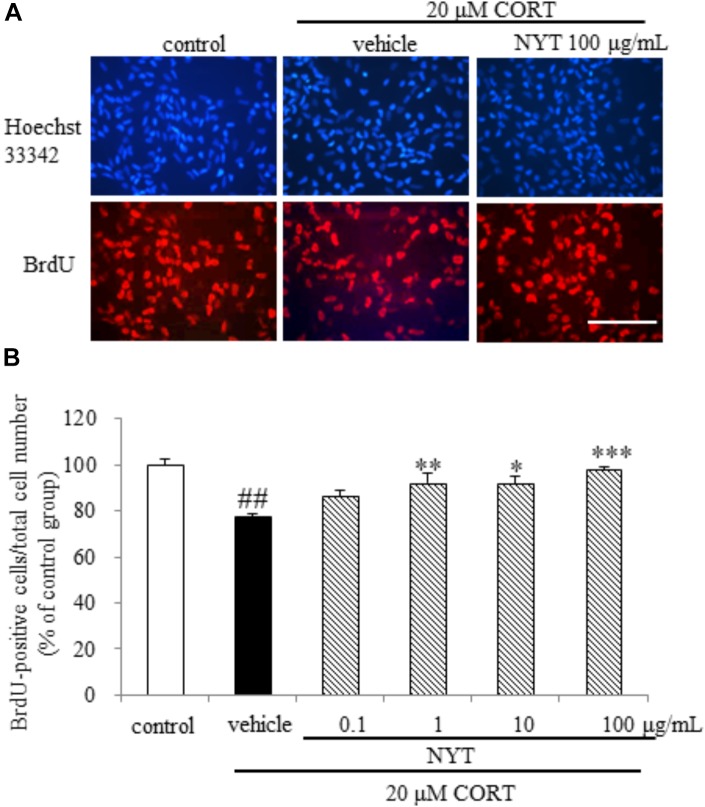
Effect of NYT on CORT-induced inhibition of hippocampal neurogenesis in an *in vitro* model. The dose response of NYT on CORT-induced inhibition of proliferation assessed using BrdU incorporation in the presence of bFGF. **(A)** Representative fluorescence microscopic images of Hoechst 33342 (blue) and BrdU (red) staining after 72-h treatment of NPCs. Scale bar = 100 μm. **(B)** Quantitative analyses of the number of Hoechst 33342- and BrdU-positive cells after 72-h treatment of cells. Data are expressed as the mean ± SEM (*n* = 4). ^##^*p* < 0.01 vs. the control group, Student’s *t*-test. ^∗^*p* < 0.05, ^∗∗^*p* < 0.01, ^∗∗∗^*p* < 0.001 vs. the vehicle-treated group, Dunnett’s test.

To examine whether NYT directly affects NPC proliferation, we isolated NPCs from the adult mouse hippocampus as described in the Materials and Methods. We found that CORT treatment reduced the ratio of BrdU-positive cells to total cells, and this reduction was inhibited by treatment with NYT in a dose-dependent manner (Figures 7A,B). These results indicate that NYT directly attenuates the reduction in NPC proliferation induced by CORT treatment.

## Discussion

Here we showed NYT treatment improved CORT-induced behavioral abnormalities and inhibition of hippocampal neurogenesis.

Depressive disorder is characterized by low mood and/or anhedonia, combined with several cognitive and vegetative symptoms. Chronic stress and HPA-axis dysfunction are generally considered risk factors for the development of psychiatric disorders, including major depression. In addition, stress and stress hormone also impair hippocampal-dependent forms of memory in humans and mice (de Kloet et al., 1999). For example, the administration of stress levels of cortisol to normal human subjects selectively impairs verbal declarative memory without affecting non-verbal memory (Newcomer et al., 1994). Furthermore, a previous study showed that the depressive-like behaviors induced by chronic CORT treatment lasted for at least 3 weeks after treatment finished (Shibata et al., 2015). For these reasons, chronic CORT treatment appears to model a depressive-like state and the memory disruption observed in patients with depression, and the CORT-induced depression model in rodents is considered to be suitable for evaluating the therapeutic effect of drugs for depression. In accordance with a previous study, long-term CORT treatment continued to affect the behavior of mice for 4 weeks after completion of the treatment in the present study. We also showed that NYT treatment increased the latency to immobility in both the tail suspension test and forced swim test, and ameliorated the CORT-induced increase in immobility time in the tail suspension test, but not the forced swim test. We also showed that NYT treatment improved the sucrose consumption rate in the sucrose preference test (Figure 3). The latency to immobility has been used to detect the efficacy of fluoxetine, antagonists of glutamate receptors, and cytidine in rats (Contreras et al., 2001; Carlezon et al., 2002; Padovan and Guimarães, 2004), and psychostimulants, such as amphetamine, were reported to have no effects on the duration or latency to immobility in the forced swim test (Castagné et al., 2009). These results suggest that NYT has therapeutic effects on depressive-like symptoms in the CORT-induced depression model. The previous report also showed that fluoxetine treatment affected only the latency but not the duration of immobility in C57BL/6 mice in the forced swim test (Castagné et al., 2009). In another report, fluoxetine was reported to ameliorate the CORT-induced increase in immobility time duration in the tail suspension test but not in the forced swim test (Sawamoto et al., 2016). NYT treatment was previously reported to increase serotonin content in the cerebral cortex and substantia nigra in olfactory bulb lesion mice (Song et al., 2001). Therefore, the mechanism of action of NYT in the CORT-induced depression model might be similar to that of fluoxetine, in part. Furthermore, we showed that NYT treatment improved CORT-induced memory disruptions in the Y-maze and novel object recognition tests, without affecting locomotor activity (Figures 2, 4).

The selective serotonin reuptake inhibitors (SSRIs) and serotonin-norepinephrine reuptake inhibitors are widely used for first-line treatment of depressive disorders. The efficacy of these drugs led to the monoamine hypothesis of depression, which postulates a pathophysiological role of decreased monoamine levels in depression. However, SSRIs do not show beneficial effects until 2 weeks and the effects do not peak until 6–9 weeks after the start of treatment (Artigas et al., 1996; Gardier et al., 1996). In this regard, the requirement for hippocampal neurogenesis for the effects of antidepressants has been investigated using X-irradiation to disrupt hippocampal neurogenesis in the rodent brain (Santarelli et al., 2003). According to previous reports, the dependence of the behavioral effects of antidepressants on neurogenesis is affected by many factors, such as the genetic background of the animals, nature of the antidepressant, and type of behavioral paradigm. In C57BL/6 mice, the behavioral effect of fluoxetine is dependent on hippocampal neurogenesis in the novelty-suppressed feeding test, but not in the forced swim test (David et al., 2009). Since CORT crosses the blood-brain barrier, and the hippocampus is enriched with corticosteroid receptors, certain hippocampal functions are susceptible to disruption by stress (Lucassen et al., 2015). Glucocorticoids and antidepressants have been reported to modulate adult neurogenesis in opposing directions, and hippocampal neurogenesis is required for treatment in the CORT-induced depression model. Previous reports showed that chronic CORT exposure affected the proliferation of progenitor cells in the dentate gyrus of the hippocampus, but not the survival and maturation of newborn cells in C57BL/6 mice (David et al., 2009). Here, we showed that chronic CORT treatment reduced the number of Ki67- and DCX-positive cells, but not BrdU- and GFAP-positive cells in the dentate gyrus. In addition, 4 weeks of treatment with NYT improved the reduction of Ki67- and DCX-positive cell numbers, but had no effect on BrdU- and GFAP-positive cell numbers in the dentate gyrus (Figures 5, 6). In addition, we also showed that NYT treatment ameliorated the CORT-induced inhibition of proliferation in an *in vitro* assay (Figure 7). In previous reports, chronic CORT treatment decreased the proliferating cell number at 21 days after CORT treatment started (David et al., 2009). However, CORT treatment might have no effect on the proliferating cell number before NYT treatment started because CORT treatment did not change the BrdU-positive cell number in comparison to the control group in the present study. This difference might be derived from the difference in mouse species or protocol used. Immunostaining for DCX reflects the sum of neuronal differentiation and survival of migratory young neurons born 4–14 days before staining. On the other hand, GFAP is expressed in astrocytes, and the number of newborn cells becoming GFAP-positive is only a fraction of the total pool of GFAP-positive cells. In the present study, CORT treatment affected only the DCX-positive cell number, not the GFAP-positive cell number, which might be a result of the different expression times of each marker. Therefore, the effect of NYT on the differentiation of newborn cells into neurons remains unclear in the present study. However, these results indicate that one of the effects of NYT on depressive-like behaviors might in part be related to the improvement of neurogenesis inhibition.

Adult hippocampal neurogenesis under stress is regulated by several signaling pathways activated by glucocorticoids, including the nitric oxide (NO) signaling pathway. A previous study showed that treatment with a synthetic NO synthase (NOS) inhibitor, such as N(G)-nitro-L-arginine methyl ester (L-NAME), improved depressive-like behaviors and hippocampal neurogenesis in mice, and chronic inhibition of NOS increased cell proliferation and had no effect on cell death in the dentate gyrus of the rat hippocampus (Park et al., 2003; Moreno-López et al., 2004). These results suggests that NO may inhibit cell proliferation in the dentate gyrus. There are three different forms of NOS that account for NO production; neuronal NOS (nNOS), inducible NOS (iNOS), endothelial NOS. A previous study showed that CORT treatment increased iNOS but not nNOS expression in the hippocampal dentate gyrus, and an iNOS specific inhibitor improved CORT-induced inhibition of hippocampal neurogenesis (Pinnock et al., 2007). On the other hand, NYT was reported to inhibit activation of nuclear factor kappa-light-chain-enhancer of activated B cells (NF-κB) signaling and iNOS induction induced by interleukin-1beta (IL-1 β) in hepatocytes (Tanaka et al., 2014). In addition, Panax ginseng extract, a component of NYT, was reported to exert antidepressant effects via inhibition of NF-κB activation and iNOS induction (Choi et al., 2018). Although we did not investigate the signaling pathway involved in neurogenesis, NYT might improve hippocampal neurogenesis via inhibition of iNOS induction. However, further experiments are needed to clarify the mechanisms mediating the actions of NYT.

## Conclusion

This study shows that NYT treatment improves depressive-like behaviors and memory impairments in the chronic CORT exposure model. In addition, NYT also blocks the inhibition of proliferation of hippocampal NPCs induced by CORT in *in vivo* and *in vitro* models. Our findings suggest that these behavioral improvements may be associated with increased hippocampal neurogenesis. Moreover, these results suggest that NYT may be a promising therapeutic agent for depression.

## Author Contributions

KM, NF, RT, and AI contributed to the conception and design of the study. KM conducted all experiments, analyzed the data, and wrote the manuscript. NF, RT, and AI revised the manuscript. All authors gave final approval for the version of the manuscript that has been submitted for publication.

## Conflict of Interest Statement

KM, NF, and RT are employees of Kracie Pharma, Ltd., Pharmacological department of herbal medicine is an endowment department, supported with an unrestricted grant from Kracie Pharma, Ltd. The remaining author declares that the research was conducted in the absence of any commercial or financial relationships that could be construed as a potential conflict of interest.
